# DNA methylation profiling of acute chorioamnionitis-associated placentas and fetal membranes: insights into epigenetic variation in spontaneous preterm births

**DOI:** 10.1186/s13072-018-0234-9

**Published:** 2018-10-29

**Authors:** Chaini Konwar, E. Magda Price, Li Qing Wang, Samantha L. Wilson, Jefferson Terry, Wendy P. Robinson

**Affiliations:** 1 BC Children’s Hospital Research Institute, 950 W 28th Ave, Vancouver, BC V5Z 4H4 Canada; 2 Department of Medical Genetics, University of British Columbia, Vancouver, BC V6H 3N1 Canada; 3 Department of Pediatrics, University of British Columbia, Vancouver, BC V6H 3N1 Canada; 4 University of British Columbia, Vancouver, BC V6H 3N1 Canada; 5 Department of Pathology, BC Children’s Hospital, Vancouver, BC V6H 3N1 Canada

**Keywords:** Placenta, Preterm birth, Acute chorioamnionitis, DNA methylation

## Abstract

**Background:**

Placental inflammation, often presenting as acute chorioamnionitis (aCA), is commonly associated with preterm birth. Preterm birth can have both immediate and long-term adverse effects on the health of the baby. Developing biomarkers of inflammation in the placenta can help to understand its effects and potentially lead to new approaches for rapid prenatal diagnosis of aCA. We aimed to characterize epigenetic variation associated with aCA in placenta (chorionic villi) and fetal membranes (chorion and amnion) to better understand how aCA may impact processes that lead to preterm birth. This study lays the groundwork for development of novel biomarkers for aCA.

**Methods:**

Samples from 44 preterm placentas (chorionic villi) as well as matched chorion and amnion for 16 of these cases were collected for this study. These samples were profiled using the Illumina Infinium HumanMethylation850 BeadChip to measure DNA methylation (DNAm) at 866,895 CpGs across the genome. An additional 78 placental samples were utilized to independently validate the array findings by pyrosequencing.

**Results:**

Using a false discovery rate of < 0.15 and average group difference in DNAm of > 0.05, 66 differentially methylated (DM) CpG sites were identified between aCA cases and non-aCA cases in chorionic villi. For the majority of these 66 DM CpGs, the DNAm profile of the aCA cases as compared to the non-aCA cases trended in the direction of the blood cell DNAm. Interestingly, neutrophil-specific DNAm signatures, but not those associated with other immune cell types, were capable of separating aCA cases from the non-aCA cases.

**Conclusions:**

Our results suggest that aCA-associated placentas showed altered DNAm signatures that were not observed in the absence of aCA. This DNAm profile is consistent with the activation of the innate immune response in the placenta and/or reflect increase in neutrophils as a response to inflammation.

**Electronic supplementary material:**

The online version of this article (10.1186/s13072-018-0234-9) contains supplementary material, which is available to authorized users.

## Background

Approximately 15 million children globally (10% of all live births) are born before 37 weeks of gestation and thus classified as preterm births (PTB) [1]. These babies are at an increased risk of life-threatening infections in the first few weeks of life, as well as long-term health complications [2, 3]. Preterm births are commonly associated with inflammation of the placenta and fetal membranes (amnion and chorion) known as chorioamnionitis, estimated to be present in approximately 40% of PTBs [4, 5]. Chorioamnionitis is also a risk factor for newborn complications, regardless of gestational age (GA) at birth [4, 6, 7]. Acute chorioamnionitis (aCA) is characterized by an infiltration of maternal neutrophils through the chorioamniotic membranes and into the amniotic space [8]. This acute inflammation is associated with an increased risk of fetal inflammatory response syndrome (FIRS), which is life-threatening to the newborn and may often arise from failure to suppress immune response against maternal cells that cross the placenta into fetal circulation [8]. Currently, aCA is diagnosed by assessment of clinical signs such as maternal fever, fetal tachycardia, maternal tachycardia, and uterine fundal tenderness. These clinical signs have variable sensitivity and are non-specific for aCA; thus, a more sensitive and specific test is needed for earlier and accurate detection of aCA, which in turn may improve PTB-associated outcomes.

Acute chorioamnionitis may be associated with microbial invasion of the amniotic space; however, it can also occur in the absence of detectable microorganisms and may be triggered by non-microbial “danger signals” including cellular stress and cell death [9, 10]. The fetus also mounts an inflammatory response, which together with aCA constitutes amniotic fluid infection/inflammation syndrome (AFIS) and associated with changes in the placental and circulating fetal cytokines. Acute chorioamnionitis is associated with other inflammatory lesions of the placenta including acute intervillositis and villitis, which affect the chorionic villous trees [7, 11]. Thus, characterizing these molecular or cellular changes in the placenta can in turn improve our current understanding of the full consequences of aCA.

Epigenetics may be a useful tool to characterize some of the molecular and cellular processes involved in placental inflammation, yet its role in the context of aCA is substantially understudied. Epigenetic influences refer to mitotically heritable chemical modifications to DNA or the proteins around which DNA is bound that occur in the absence of a change to the nucleotide sequence. DNA methylation (DNAm), the addition of a methyl group to the 5′carbon of cytosine, most typically at cytosine–phosphate–guanosine (CpG) dinucleotides, is a commonly interrogated epigenetic mark in human population studies. The relationship between DNAm and gene expression regulation is complex and tends to be dependent on genomic context [12]. In the context of PTB, only a limited number of studies have investigated DNAm changes in the placenta and fetal membranes [13–15]. While these studies provided some preliminary insights on DNAm patterns associated with PTB, rarely are the same CpG candidates reported as differentially methylated (DM) between cases and controls. Additionally, the definition of PTB pathology was ambiguous, which limits the utility of these studies in understanding the role of DNAm in aCA. A focus on a distinct pathology linked to PTB, such as aCA, is needed to further research in this area.

While DNAm may reflect changes to gene expression in specific cell types, it may also reflect altered cell composition. DNAm signatures differ strikingly between different cell lineages, including those in extra-embryonic tissue. Chorionic villi have a unique DNAm landscape compared with maternal decidua, fetal membranes (chorion and amnion), and embryonic tissues (brain, kidney, muscle, spinal cord) [16]. In whole placenta samples (chorionic villi), DNAm patterns also change with GA [17], and in certain placental pathologies [16]. We hypothesize that the increase in the number of immune cells that occur during placental inflammation will be reflected in changes to DNAm in whole placental samples. Specifically, we sought to address three questions: (1) Are there genome-wide DNAm changes associated with aCA in chorionic villi, chorion and amnion? (2) Are there common aCA-associated DNAm changes between chorionic villi, chorion, and amnion? (3) Can we use DNAm signatures to characterize immune cell types in aCA-associated placentas?

**Fig. 1:**
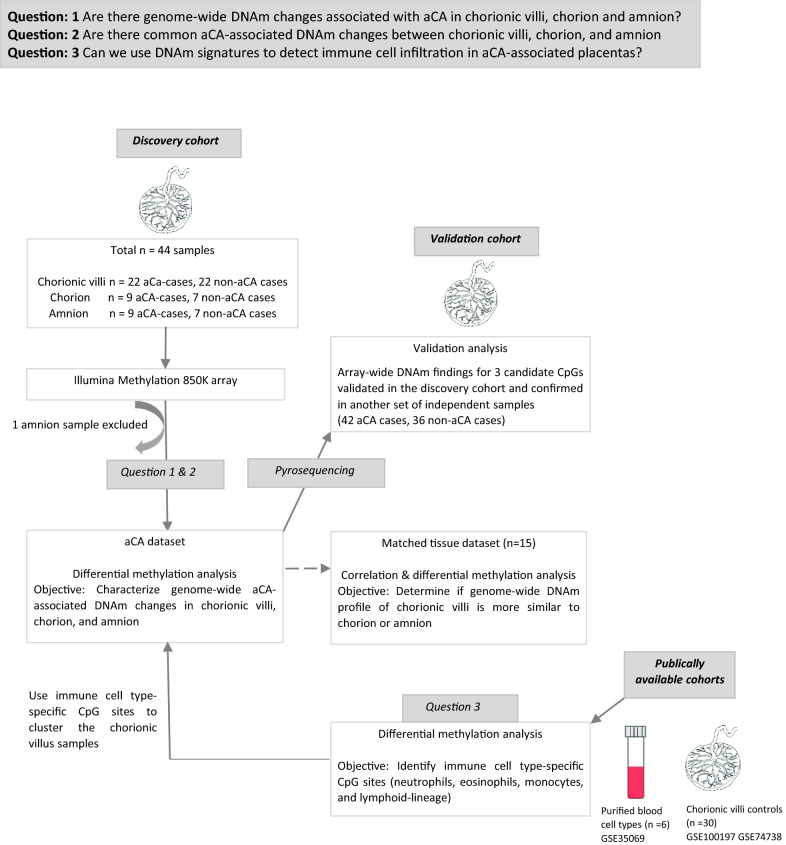
Schematic representation of the study design and workflow

An overview of the study design is presented in Fig. 1. We first compared genome-wide DNAm profiles of aCA-associated pregnancy tissues (22 placental chorionic villi, including 9 with matched chorion and amnion samples) to profiles of non-aCA preterm pregnancy tissues (22 placental chorionic villi, including 7 with matched chorion and amnion samples). Next, we identified overlapping aCA-associated DNAm changes across the three tissue types, chorionic villi, chorion, and amnion. Differentially methylated CpG sites associated with aCA in the “discovery cohort” were followed up in an independent set of samples (*N* = 42 aCA cases, 36 non-aCA cases) by a site-specific technique for measurement of DNAm (pyrosequencing). Finally, we used unsupervised hierarchical clustering with neutrophil-specific CpG sites to segregate chorionic villus samples based on aCA status. Collectively, our genome-wide array analysis of aCA placentas was able to capture some immunity-related changes, which may be in part attributable to changes to immune cell-type ratios and gene expression during placental inflammation in aCA.

## Methods

### Ethics approval and sampling

This study was approved by the research ethics committees of University of British Columbia and the Children’s and Women’s Health Centre of British Columbia (H04-70488). Samples used in this study were de-identified, and only non-identifiable clinical information was available for this study. A small piece of the fetal membranes (amnion and chorion) was removed and washed thoroughly to minimize maternal blood contamination. Independent samples of chorionic villi were taken from 2 to 3 distinct locations from the fetal side of the placenta to avoid maternal decidua. Extraction of DNA was performed by the standard salting out method [18], and DNA was assessed for purity on the Nanodrop 1000 spectrophotometer (ThermoScientific, USA).

### Sample information

Discovery cohort: Placentas from 44 PTBs, 22 with aCA and 22 without aCA were collected from the deliveries at the BC Women’s Hospital. A subset of these samples has been previously described in Wilson et al. [19]. Diagnosis of aCA was performed using validated histological criteria by clinical pathologists [20]. Distribution of non-aCA preterm cases was chosen to be of mixed etiology based on clinical history and placental examination and is as follows: 11 cases of spontaneous premature preterm rupture of the membranes, 6 cases of placental abruption, 2 cases of placental previa, 1 case of preterm labor, 1 case of preeclampsia, and 1 case of unexplained PTB. Exclusion criteria for samples were (1) chromosomal abnormalities either diagnosed prenatally by amniocentesis or on the placenta postnatally after delivery as determined by postnatal screening, (2) fetal malformations, (3) intrauterine growth restriction, and (4) multi-fetal pregnancies. Demographic and clinical characteristics of the discovery cohort are presented in Table 1 and Additional file 1: Table S1. Although the GA at delivery differed between the groups, the age range was the same and GA was accounted for in statistical analyses.

**Table 1:** Demographic and clinical characteristics of the discovery cohort

Variables	aCA (*n* = 22)	Non-aCA (*n* = 22)	*p* value
Maternal age, years (mean)	19.6–43.9 (33.41)	20.4–43.5 (32.08)	ns
GA at delivery, weeks (mean)	28–36 (30.74)	28–36.7 (32.28)	< 0.01
Birth weight (SD)	− 1.02 to 1.51	− 1.36 to 0.73	ns
Fetal sex (*M*/total)	11/22	13/22	ns

*p* values are calculated by Wilcoxon–Mann–Whitney rank sum test for continuous variables, Fisher’s exact test for fetal sex

*ns* non-significant

Since ancestry was largely unknown in the discovery cohort, a panel of 57 ancestry informative marker (AIM) single nucleotide polymorphisms (SNPs) [21–23] was used to evaluate population stratification. These were assessed by the Sequenom iPlex Gold platform (Génome Québec Innovation Centre, Montréal, Canada). Ancestry was inferred from AIM SNPs using multi-dimensional scaling (MDS) based on the method as described in Del Gobbo et al. [24]. While overall similar, there were significant differences in the distribution of ancestry MDS coordinates between aCA cases and non-aCA cases as assessed by Kolmogrov–Smirnov (KS) tests; therefore, ancestry was accounted for in statistical analyses.

Validation cohort: Fresh frozen placental chorionic villi was obtained from the Anatomical Pathology Laboratory at BC Children’s & Women’s Health Centre. Clinical characteristics of the validation cohort are presented in Table 2 and Additional file 1: Table S2. Both aCA cases and non-aCA preterm cases were chosen using the same criteria as the discovery cohort. DNA was extracted using the same protocol as described for the discovery cohort samples.

**Table 2:** Demographic and clinical characteristics of the validation cohort

Variables	aCA (*n* = 42)	Non-aCA (*n* = 36)	*p* value
Maternal age, years (mean)	21–44 (32)	17–43 (29.27)	< 0.03
GA at delivery, weeks (mean)	20–41 (26.83)	20–39 (29.2)	< 0.02
Birth weight (SD)	− 2.24 to 1.23	− 3.13 to 1.67	ns
Fetal sex (*M*/total)	24/42	21/36	ns

*p* values are calculated by Wilcoxon–Mann–Whitney rank sum test for continuous variables, Fisher’s exact test for fetal sex

*ns* non-significant

### Illumina Infinium HumanMethylationEPIC BeadChip quality control and preprocessing

Chorionic villi (*n* = 44), amnion (*n* = 16), and chorion (*n* = 16) from the discovery cohort samples were run on the Illumina Infinium HumanMethylationEPIC BeadChip (850K array), which quantifies DNAm at 866,895 CpG sites across the genome [25]. To minimize technical effects of sample processing, all samples were run in the same batch and by the same operators, and 4 samples were run in duplicate to assess data processing. See Additional file 2: Figure S1 to view distribution of samples across chips.

The discovery cohort genomic DNA was purified using DNeasy blood and tissue kit (Qiagen, CA, USA) followed by bisulfite conversion of the purified DNA using the Zymo EZ DNA Methylation™ Kit (Zymo Research, USA). Bisulfite converted DNA was then whole-genome amplified, enzymatically fragmented, and hybridized to the array as per the 850K array protocol [25]. Chips were scanned using an Illumina HiScan2000, and raw intensity was read into GenomeStudio Software (Illumina). Within Genome Studio samples were background normalized, after which data were read into R statistical software (version 3.4.1) with the Bioconductor *lumi* [26] package to generate M values from the signal intensities.

An initial sample quality control check (QC) was performed in GenomeStudio using Illumina’s 636 control probes to assess technical parameters including array staining, extension, hybridization, target removal, specificity, and bisulfite conversion. Clustering of samples based on all probes confirmed tissue identity except for one amnion and one chorion sample which were likely tissue label swaps as they were from the same placenta and clustered with the wrong fetal membrane tissue group. The samples were reassigned labels and were kept in for the remainder of the analysis. One amnion sample clustered further away from the amnion group as seen in hierarchical clustering and PCA (Additional file 2: Figure S2a and S2b) and was removed from the analysis as it is likely this is due to either poor DNA quality or a contaminated sample. Sample quality was further assessed as in Price et al. [27], and no other sample was excluded from the analysis.

After initial QC, 44 placental chorionic villi, 16 chorion, and 15 amnion samples were identified for the aCA analysis. Further analysis was conducted independently for the three tissues. Probe filtering was performed as shown in Additional file 1: Table S3 [28–30]. To account for type I–type II probe differences on the 850K array, functional normalization [31] was done. ComBat [32] was used to correct for known technical variation associated with Sentrix_row (chip position) and Sentrix_Id (chip Id). Correlation of the technical replicates in chorionic villi improved from the raw data to batch corrected cleaned data (Additional file 2: Figure S3). Because there were no technical replicates for amnion and chorion, performance metrics in R *wateRmelon* Package [33] were used to monitor data preprocessing for the fetal membranes.

### Differential methylation analysis of acute chorioamnionitis data

To identify differential methylation associated with aCA, we first used a candidate gene approach. Genes of interest (*n* = 12) were chosen based on publications reporting a genetic association with chorioamnionitis [34–36], placental inflammation [37, 38], and neonatal sepsis/infection [39–41]. CpG sites from the 850K array that mapped to these genes were then identified (195 CpG sites, Additional file 1: Table S4). Using this set of biologically relevant CpGs, we modeled DNAm as a function of aCA status with GA, fetal sex, and ancestry included as additive covariates. Subsequently, an epigenome-wide association study (EWAS) was conducted using the batch corrected cleaned data (711789 CpG sites). To account for multiple tests, the resulting *p* values were adjusted using the Benjamini and Hochberg [42] false detection rate (FDR) method. Group differences in DNAm (Δ*β*) were then calculated by subtracting the average beta (*β*) of non-aCA cases from aCA cases on an individual CpG site basis. Differentially methylated sites associated with aCA were identified based on FDR and Δ*β* thresholds. Using similar thresholds, sites identified in the chorionic villi were also examined in the chorion and amnion samples and the overlap of the chorionic villi sites in chorion and/or amnion was confirmed by a random sampling function as described in Wilson et al. [19]. Finally, ‘dmrFind’ function in the R *charm* package was used to find differentially methylated regions (DMR) [43], as described in [27].

### Pyrosequencing

We performed pyrosequencing for some of the CpG sites identified as DM in the 850K array comparison of aCA cases and non-aCA cases. Genomic DNA was bisulfite converted using the EZ DNA methylation Gold kit (Zymo, USA) as per manufacturer’s protocol. PyroMark Assay Design software version 2.0 was used to design the forward, reverse, and sequencing primers. Primer sequences and reaction conditions for all pyrosequencing assays are listed in Additional file 2: Figure S4. For quality control, synthetic fully methylated and unmethylated samples (standard controls,) were included on each plate. PyroMark Q-CpG software (Qiagen) was used to generate quantitative methylation levels of the targeted CpG of interest. Spearman’s rank-order correlation was used to illustrate agreement between the 850K array findings and pyrosequencing data. A linear model was fitted for each follow-up CpG site, with aCA status as main effect and GA, fetal sex, and ancestry as covariates.

### Correlation analysis and differential methylation analysis on matched tissue dataset

To address the overlap between differential methylation for different tissues, it is useful to understand patterns of correlation across our three aCA-associated tissues (chorionic villi, chorion, amnion). To do this, we utilized the strength of our matched sample cohort of 15 individuals. Data preprocessing of the matched tissue cohort was performed as described in Additional file 3: Methods S1. Correlation of DNAm on a per CpG level was calculated between chorionic villi and the fetal membranes independently using Spearman rank-order correlation on *M* values. Adopting a similar approach from [29], we generated a null distribution of correlations by shuffling the order of the chorionic villus samples and ran the correlations calculations with each fetal membrane. Comparing against the null distribution allowed us to confirm whether the correlated sites between chorionic villi and fetal membranes were significantly more than what we would expect by chance.

To quantify the similarity between fetal membranes and placental chorionic villi, differential DNAm analysis on a per CpG level was also performed between the tissue pairs (chorionic villi-chorion and chorionic villi-amnion) by applying a linear model to *M* values using *limma* [44]. This is described in detail in Additional file 3: Methods S2, and Additional file 1: Table S5.

### Immune cell-type analysis on the acute chorioamnionitis dataset

Because we hypothesized that altered DNAm exhibited by aCA-associated placentas might reflect an increase in the number of immune cells, we anticipated that the aCA cases and immune cell types might follow similar trends in DNAm for the DM sites identified in our EWAS analysis. To test this, we first utilized a publicly available dataset assessing DNAm in blood immune cell types isolated from six healthy adults [45]. These samples had been run on the Illumina Infinium HumanMethylation450 BeadChip (450K array), measuring DNAm at 485,512 CpG sites across the genome [46], 93.3% of which overlap the 850K array used in the present study. Beta value distributions of DM sites were compared across aCA cases and the blood immune cell types to identify concordance in DNAm patterns.

To identify immune cell-type-specific DNAm, we used the previously described publicly available dataset [45] and also included 30 chorionic villi controls from two published studies in our laboratory [19, 47]. Similar site filtering criteria were adopted as described previously in Table S3. To identify neutrophil-specific CpG sites, we first performed differential methylation analyses for the following comparisons: (1) neutrophils–chorionic villi, (2) neutrophils–eosinophils, (3) neutrophils–monocytes, and (4) neutrophils–lymphoid lineage cells (T cell + B cells + Natural Killer cells), using linear modeling on filtered and normalized data (442,495 CpGs). We next identified overlapping DM CpG sites between the above comparisons to determine neutrophil-specific CpG sites. This procedure was also used to identify eosinophil-specific CpG sites, monocyte-specific CpG sites, and lymphoid lineage-specific CpG sites. Finally, these immune cell-type-specific CpG sites were used to cluster the chorionic villus samples in the discovery cohort. Stability of the resulting clusters was determined using the R package *pvclust* [48] and the R *sigClust2* package [49] was used to assess if the resulting clusters were significantly different from one another, both using 1000 iterations.

## Results

### Characterization of unique aCA-associated DNAm changes in chorionic villi and fetal membranes

**Fig. 2:**
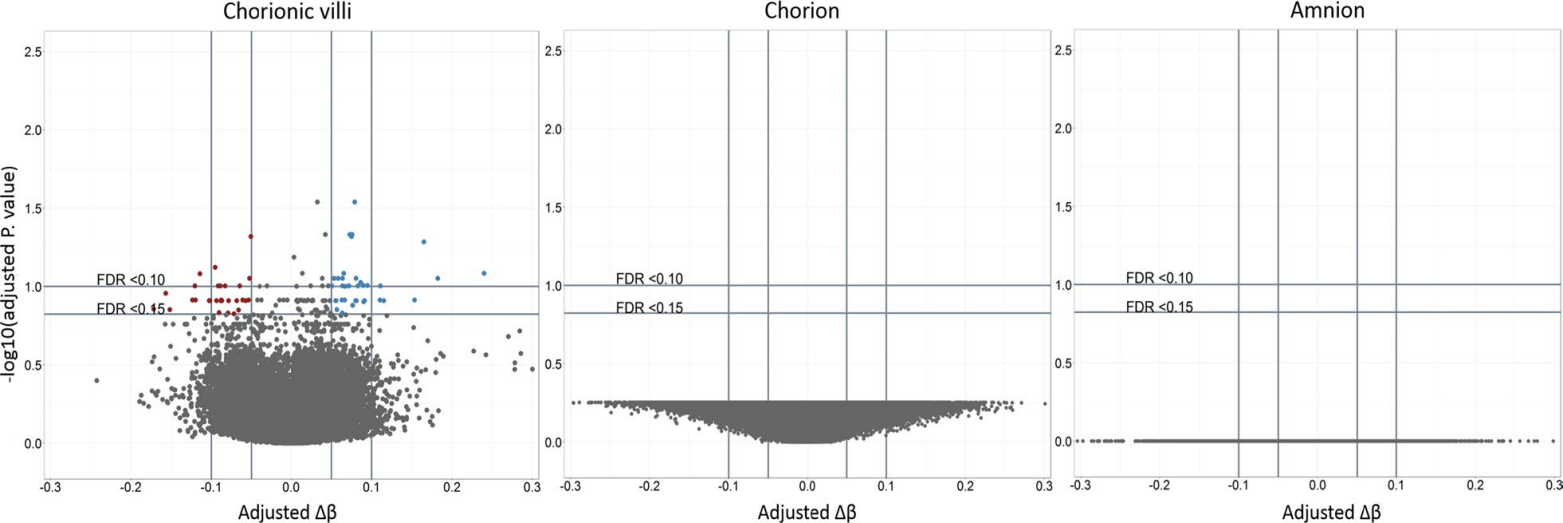
Acute chorioamnionitis array-wide volcano plots. For each probe, FDR-corrected *p* values from fitted linear models were plotted against group differences in DNAm for each tissues. Sites at FDR < 0.15 and adjusted Δ*β* > 0.05 are highlighted in red and blue. Sites highlighted in red are those that are hypermethylated in aCA cases compared to non-aCA cases. Sites highlighted in blue are those that are hypomethylated aCA cases compared to non-aCA cases. The flat volcano plots demonstrate a lack of differential methylation associated with aCA in the fetal membranes after correction for multiple comparisons

To investigate differences in DNAm between aCA cases and non-aCA cases, we fitted a linear model testing for differential methylation in each of the three tissues in the discovery cohort, chorionic villi (22 aCA, 22 non-aCA), amnion (8 aCA, 7 non-aCA), and chorion (9 aCA, 7 non-aCA). We first looked at DNAm in a subset of 12 genes that might be biologically relevant to aCA (Additional file 1: Table S3). CpG sites from the DNAm array that mapped to these candidate genes were not DM between aCA cases and non-aCA cases. Next, using an epigenome-wide approach, no sites were identified as DM in amnion or chorion, but 66 sites were DM in chorionic villi (FDR < 0.15 and Δ*β* > 0.05) (Fig. 2). Detailed descriptions of these sites are provided in Additional file 1: Table S6. We chose less stringent statistical cutoffs here as our small sample size may limit our ability to detect significant DNAm differences at individual CpG sites. Hierarchical clustering of chorionic villus samples using these 66 DM CpG sites completely separated the aCA cases from the non-aCA cases (Additional file 2: Figure S5). Although non-significantly enriched for any gene ontology terms using ermineJ [50], some of the DM sites were located within immune-relevant genes such as *HLA*-*E*, *CXCL14*, *RAB27A, IRX2,* and *HSD11B2*. Each of these DM CpG sites were located 200-1500 base pairs upstream of the transcription start site.

We also tested for differences in DNAm across regions (i.e., DMRs) to integrate information across neighboring CpGs. This reduces the number of statistical tests, potentially increasing the power to detect a DNAm change. However, none of the DMRs withstood correction for multiple tests in each of the three tissues (chorionic villi, chorion, and amnion).

Male fetuses are at an increased risk of PTB [51, 52] and are more likely to show adverse outcomes including chronic inflammation, respiratory distress syndrome, neonatal sepsis, infection, and stillbirth [53–56]. To identify whether there may be sex-specific DNAm changes associated with placental inflammation, we repeated the EWAS analysis on male and female chorionic villus samples separately. We fitted a linear model testing for differential methylation in the discovery cohort of chorionic villus samples, analyzing males (11 aCA, 13 non-aCA) and females (11 aCA, 9 non-aCA) separately. None of the 711,789 CpG sites was DM between aCA cases and non-aCA cases in males or females (FDR < 0.15 and Δ*β* > 0.05) (Additional file 2: Figure S6). However, our ability to characterize sex-specific significant differences at individual CpGs is limited by the small sample sizes.

### Pyrosequencing validation of aCA-associated differentially methylated sites

**Fig. 3:**
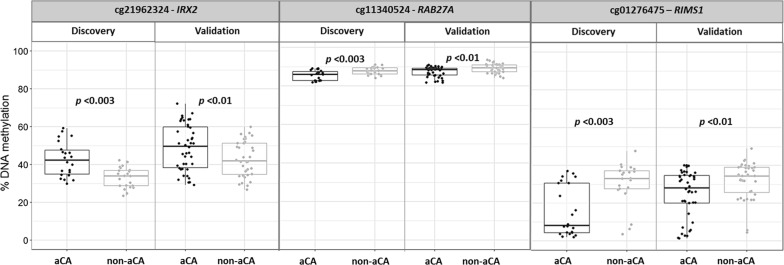
Pyrosequencing of cg11340524, cg21962324, and cg01276475 in chorionic villi. Pyrosequencing was performed to follow up differential methylation at three CpG sites (cg11340524, cg21962324, and cg01276475), identified in the chorionic villus samples in discovery cohort. We confirmed significant differential DNAm in the discovery cohort (*p *< 0.003) and replicated in the independent set of samples (i.e., validation cohort) (*p* < 0.01)

To verify our array-wide findings, we performed pyrosequencing on three of the DM CpG sites (cg01276475, cg11340524, and cg21962324) in chorionic villi in the discovery cohort (22 aCA cases, 22 non-aCA cases) and in an additional set of 42 aCA and 36 non-aCA cases (i.e., validation cohort). One CpG site, cg01276475 (*RIMS1*) was chosen as it showed highest magnitude of methylation difference (∆*β* > 0.20) between the groups in the discovery cohort. The other two sites (cg11340524, *RAB27A*; cg21962324, *IRX2*) were chosen to follow up based on functional relevance in immune system and inflammation, which may be of interest in aCA. DNAm measured by pyrosequencing was significantly correlated with the 850K array data at all three CpG sites [cg01276475, *r* = 0.8 (*p *< 2.2e−16); cg21962324, *r* = 0.8 (*p *< 2.2e−16); cg11340524, *r* = 0.7 (*p *< 1.322e−07)] and was verified in the discovery cohort (*p *< 0.003). Furthermore, the differences at these three CpG sites were also confirmed in the validation cohort (*p *< 0.01) (Fig. 3).

Next, we tested for differential methylation at the three selected CpG sites in males and females separately, as correcting for a large number of CpG sites may have limited our power to detect array-wide sex-specific DNAm differences between our study groups. The three DM CpG sites showed a DNAm trend in the same direction in both males and females as observed in the discovery cohort and validation cohort chorionic villus samples (Additional file 2: Figure S7). For these selected CpG sites, we also assessed variation in DNAm between fetal sexes over gestation. Though we failed to observe a significant difference in DNAm between the sexes, we did, however, observe sex-specific trends in DNAm across gestation for cg21962324 (*IRX2*) and cg01276475 (*RIMS1*) (Additional file 2: Figure S8).

### Characterization of common aCA-associated DNAm changes between chorionic villi and fetal membranes

As the aCA changes seemed limited to chorionic villi, despite that diagnosis of aCA is based on inflammation of the fetal membranes, we wanted to further understand the relationship between these tissue types. DNAm patterns vary widely by tissue type [57, 58]. Because chorionic villi and chorion are considered to partially share a trophectoderm-derived cell lineage [47], we predicted that DNAm in healthy tissues might be more similar between chorionic villi and chorion and as a consequence, DNAm changes in response to inflammation might have more overlap between chorionic villi and chorion than compared to amnion. We first confirmed that the genome-wide DNAm profile of chorionic villi is more correlated to chorion than amnion (Additional file 2: Figure S9). Next, we compared the DNAm landscapes of chorionic villi to the fetal membranes by a differential DNAm analysis. To enrich for tissue-specific DNAm differences, we adopted strict statistical (FDR < 0.01) and biological thresholds (Δ*β* > 0.20) and identified more DM sites when comparing chorionic villi to amnion than when comparing chorionic villi to chorion (Additional file 2: Figure S10). We then used the 66 DM sites in chorionic villi and fitted a linear model to test for similar changes in DNAm at these sites in the chorion and/or amnion. At the same thresholds (FDR < 0.15 and Δ*β* > 0.05), no sites were DM in amnion, while ~ 20% (13/66) of chorionic villi sites were also DM in chorion, higher than expected by chance (*p *= 0.0001). This suggests that chorion may show a somewhat overlapping response to infection as compared to chorionic villi; this may have been missed in the 850K array analysis because we were underpowered with the smaller sample size in amnion and chorion.

### Characterization of immune cell types in aCA-associated placentas (Question 3)

The majority of the aCA samples in our study were also diagnosed with acute villitis, i.e., inflammation with histological evidence of the presence of neutrophils within the chorionic villi. We therefore hypothesized that some of the aCA-associated DNAm changes captured by the 850K array could be attributable to an increase in immune cell number, especially neutrophils during placental inflammation. Therefore, we expected the 66 DM sites associated with aCA in chorionic villi may trend in the same direction as DNAm associated with blood-derived immune cell types. To test whether the aCA-associated DNAm changes were driven by changes in immune cells ratios in the placenta, we first sought to compare whether the DNAm profile of the aCA chorionic villi cases shifted in the same direction as the blood immune cell types. Specifically, when aCA chorionic villi cases were hypermethylated or hypomethylated compared to the non-aCA chorionic villi cases, we anticipated the blood immune cell types will show similar DNAm patterns compared to the non-aCA chorionic villi cases.

**Fig. 4:**
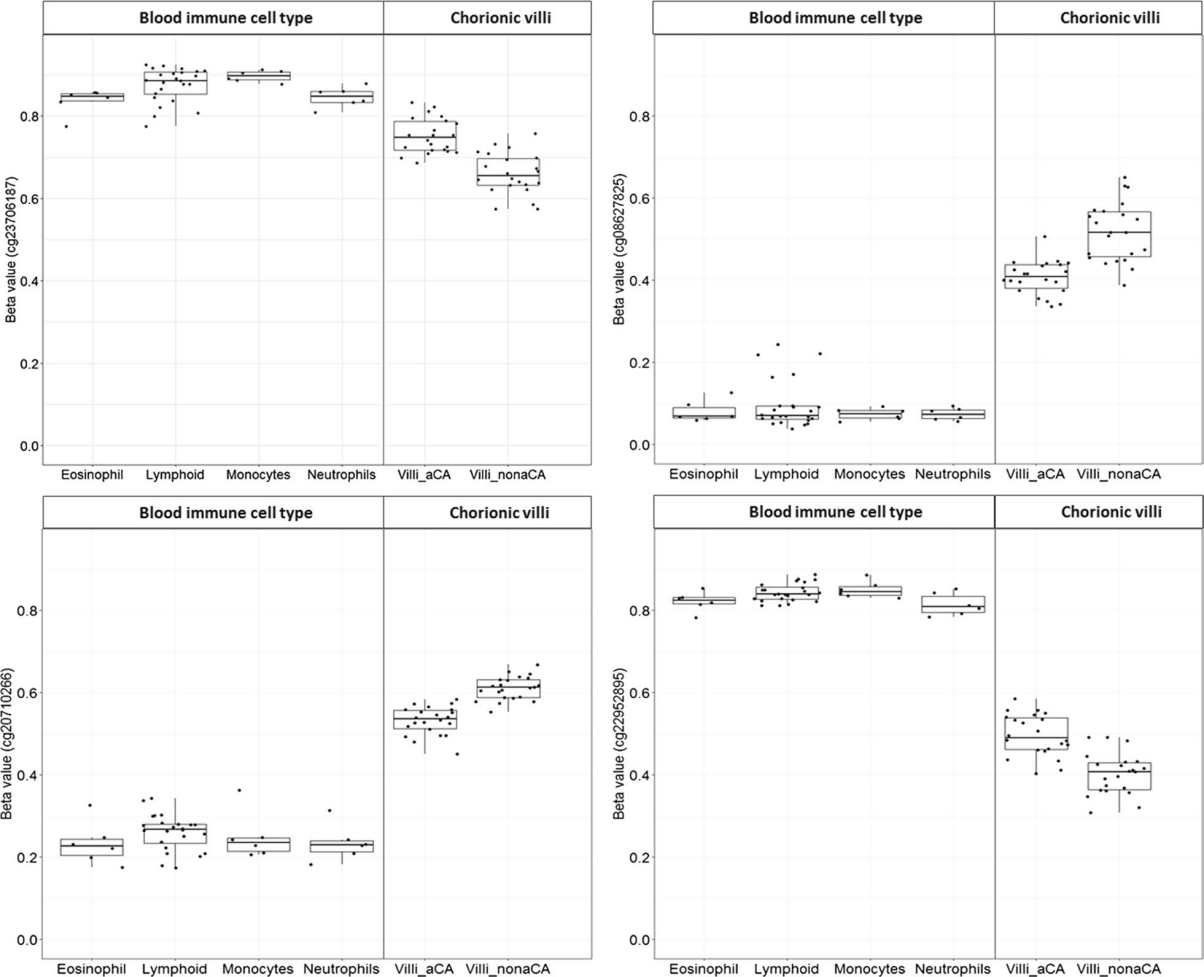
Differentially methylated CpG sites in the chorionic villi comparison of aCA cases to non-aCA cases. Box plots of DNA methylation for four representative CpGs sites that are either hypermethylated or hypomethylated in the aCA cases compared to non-aCA cases in chorionic villi. These sites are among 24/36 sites that showed similar trends in DNAm for the multiple immune cell types (eosinophil, neutrophil, monocytes, and lymphoid) as aCA chorionic villi cases. For example, when aCA cases were hypomethylated compared to non-aCA cases, immune cells were also hypomethylated compared to non-aCA

DNAm data for neutrophils, eosinophils, monocytes, lymphoid cells, and whole blood were obtained from GEO (GSE35069). As these data were run on a previous version of the Illumina array, the 450K array, only data for 36 of our 66 DM sites were available. We compared the *β* value distributions of 36/66 DM sites available in GSE35069 for each immune cell type to our data. Taking neutrophils as an example, 24/36 (66.6%) DM sites showed a similar trend in DNAm to the aCA cases versus non-aCA cases, which is higher than what we would expect by chance (*p *= 0.00001). This pattern was also observed across all the other immune cell types including whole blood, suggesting that the DNAm profile of the aCA chorionic villi cases may be more shifted in the direction of blood immune cell type than a non-aCA-associated placenta (Fig. 4).

**Fig. 5:**
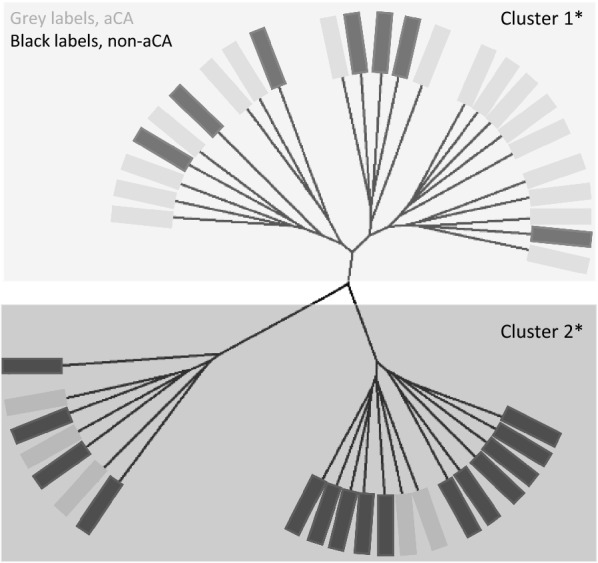
Sample clustering based on array-wide neutrophil-specific CpG sites. Euclidean clustering of our chorionic villi DNA methylation data (*n* = 44 samples) using 2069 neutrophil-specific CpG sites largely separated aCA cases from the non-aCA cases. Asterisk indicates stable and significantly different clusters as determined by pvclust and sigclust2 packages

As we only expected neutrophils and not other blood cells to be increased in the placental samples, we next sought to detect whether there was a specific immune cell type more likely to be linked to aCA-associated placentas. We identified immune cell-type-specific CpG sites using this same data by a differential methylation analysis (see Methods). Unsupervised hierarchical clustering was performed in the discovery cohort of chorionic villus samples using these immune cell-type-specific CpGs. Neutrophil-specific sites (2069 CpGs) produced two stable and significantly different clusters (Fisher’s test, *p *< 0.005) which separated most aCA cases from the non-aCA cases (Fig. 5), while the same analysis using eosinophils, monocytes, lymphoid lineage-specific sites did not yield stable clusters. No other differences were observed between the two neutrophil-specific clusters based on the available clinical information (Table 3). Neutrophil-specific cluster 1 was significantly enriched for aCA cases (*p *< 0.005), suggestive of a “placental inflammation-mediated” phenotype in this group. Neutrophil-specific cluster 2 consisted predominantly of non-aCA cases. Although two sub-clusters were observed in this latter group, the sub-clusters were not stable as determined by multiscale bootstrap resampling (1000 permutations).

**Table 3:** Clinical information on samples assigned to cluster 1 and cluster 2 obtained by neutrophil-specific CpGs

Neutrophil-specific	Cluster 1 (*n* = 22)	Cluster 2 (*n* = 20)	*p* value
aCA status (aCA/total)	17/22	5/20	< 0.005
Fetal sex (*M*/total)	15/24	9/20	ns
Gestational age, weeks	28.3–36	28–36.7	ns
Maternal age, years	21.2–43.9	20.4–43.5	ns
Fetal birth weight (SD)	− 1.14 to 1.58	− 2.46 to 0.73	ns

*p* values are calculated by Wilcoxon–Mann–Whitney rank sum test for continuous variables, Fisher’s exact test for fetal sex and aCA status

*ns* non-significant

Because we did not observe an overlap of DM CpG sites between the 66 DM aCA-associated CpGs and the neutrophil-specific CpGs, the later may manifest as subtle changes that do not meet our criteria of Δ*β* > 0.05. It is also likely that some of the stronger aCA-associated DNAm changes might reflect alterations in cell populations inherent to the placenta such as Hofbauer cells, trophoblast, and/or endothelial cells or to changes in gene expression, within some of those cell types. However, it is important to note that the 66 DM CpGs were identified using the 850K array whereas the neutrophil-specific CpGs were characterized using the 450K array, and only 36/66 DM CpG sites were common between the arrays. Additionally, different statistical and biological thresholds were used to identify the 66 DM CpGs and the neutrophil-specific CpGs, which could explain the lack of an overlap between the CpG sites.

## Discussion

To our knowledge, this is the first study to investigate genome-wide aCA-associated DNAm alterations in the placenta and fetal membranes. Our EWAS identified 66 DM CpG sites (FDR < 0.15 and Δ*β* > 0.05) associated with aCA in chorionic villi. Among the 66 were a number of CpGs located in inflammation-related genes including *HLA*-*E*, *CXCL14*, *RAB27A, IRX2,* and *HSD11B2*. Expression of *HLA*-*E* molecules in extravillous trophoblast cells of the placenta inhibits the cytotoxic activity of maternal natural killer (NK) cells, resulting in immune tolerance to the fetus during pregnancy [59]. *CXCL14* is highly expressed in the placental trophoblast cells and shown to regulate trophoblast invasion during pregnancy [60]. This chemokine acts as a selective chemoattractant for dendritic cells, monocytes, NK cells, and neutrophils, and facilitates leukocyte recruitment under different pathophysiological conditions [61, 62]. Furthermore, decreased expression of *CXCL14* was reported in spontaneous PTB placentas [63]. *RAB27A* plays a vital role in the regulation of multiple neutrophil functions such as chemotaxis, adhesion to activated surfaces, and exocytosis of granules with anti-microbial properties [64]. Transcription factor *IRX2* has been shown to control chemokine expression in breast cancer cells [65]. *HSD11B2*, on the other hand, is regulated by proinflammatory cytokines and may influence the inflammatory microenvironment [66]. Additionally, decreased expression of *HSD11B2* in chorioamnionitis-associated placentas has been reported [67]. Overall, our DNAm EWAS study provides an improved understanding of epigenetic changes that occur during placental inflammation, which is an important precursor for developing markers for earlier clinical diagnosis of aCA.

Currently, clinical examination is the routinely used method for diagnosing aCA, but assessment of clinical signs is neither sensitive nor specific. Histologic examination of the placenta is more sensitive and specific for identifying aCA, but is only possible after delivery. There has been some promise in utilizing maternal serum biomarkers to predict placental inflammatory responses, including C-reactive protein and cytokines [68–70]. However, conflicting reviews on the clinical utility of these maternal serum markers raises concerns on their reliability, sensitivity, and specificity [71–73]. Limited studies have identified altered gene expression in candidates such as *TLRs*, and *CXCLs* in chorioamnionitis-affected placentas [74–77]. Some of these inflammation-related mRNA changes were also observed during normal spontaneous labor [78], and therefore may not represent unique mRNA changes in placental inflammation.

DNA methylation is associated with gene expression, but is relatively stable, and more likely to retain a “memory” of earlier in utero exposures. In our study, we were successful in confirming some of our array-wide DNAm findings by an alternate technology and validating these DNAm results in an independent set of samples. These DNAm alterations may be useful in the development of biomarkers for rapid prenatal detection of aCA if they are also detectable in maternal blood [79], as has been shown for other conditions including cancer [80–83] and preeclampsia [84, 85]. However, the aCA-associated methylation changes reported in this study were small in magnitude and may require highly sensitive detection methods. On the other hand, a subset of aCA-associated placentas may exhibit larger DNAm changes due to increased severity of the inflammatory processes associated with aCA. It is also important to recognize that the distribution of inflammatory lesions associated with aCA is heterogeneous across the placenta, and depending on the sampling site, changes in DNAm will likely vary from subtle to large effects.

In recent years, there has been increasing interest in DNAm EWAS studies about “cell-type correction.” Differences in DNAm identified using whole tissue samples such as chorionic villi may represent a change in DNAm limited to one cell type or suggest a difference in the proportion of cell types between disease groups. These differences in cell-type proportions are often thought to be a confounding influence, and thus variance due to altered cell-type proportions is removed using computational approaches [86]. In some conditions such as in aCA, alterations in cell-type proportions may be biologically relevant to the disease pathogenesis, and therefore adjusting for cell composition will significantly reduce the variability due to the pathology, as discussed by Lappalainen and Greally [87]. Numerous studies have documented aCA-associated cellular changes primarily in the context of a microbial invasion that ascends from the lower genital tract. Invasion of the amniotic cavity stimulates a strong inflammatory response in the mother and fetus, and an increase in the concentrations of proinflammatory cytokines can be detected in the amniotic fluid [88–90]. In response to this chemotactic gradient, maternal neutrophils migrate toward the chorionic plate of the placenta. Additionally, placental tissue from cases of aCA exhibits alterations in the number of placental-specific macrophages called Hofbauer cells [91, 92]. Placental trophoblast cells also mount a specific innate immune response in the presence of a microbial infection [93–95]. In our study, the DNAm changes associated with aCA may  reflect an increase in immune cell number such as neutrophils and/or represent changes in placental cell populations as a response to inflammation such as increased secretion of *IL8* by placental trophoblast cells reported during chorioamnionitis [96]. It is also possible that some of the DNAm changes occurring in the placental trophoblast cells could overlap with the neutrophil-specific changes as these are also a part of the innate immune system. Further, we identified some common aCA-associated DNAm changes between chorionic villi and chorion, and none of the DNAm changes overlapped between chorionic villi and amnion. Because chorionic villi and chorion share a trophectoderm-derived cell lineage, the overlapping DNAm changes in these two tissues may reflect similar epigenetic response to aCA-associated inflammation. Further, shared changes between chorionic villi and chorion could also result from a similar alteration in immune cell ratios in chorionic villi and chorion.

We utilized a subset of our matched samples to gain a comprehensive understanding of DNAm landscapes in the placenta and fetal membranes. Our group previously reported on genome-wide tissue-specific DNAm patterns in the placenta, and fetal membranes [16]. However, the present study included a larger sample size and matched tissue samples. The DNAm landscape of chorionic villi was more similar to chorion than amnion, possibly because the ectodermal layer of chorion and the outer trophoblast layer of the chorionic villi are both derived from the trophectoderm. In contrast, similarities between chorion and amnion may also occur as the mesenchyme layers of both the chorion and amnion membranes have a common origin from the epiblast.

Our findings should be interpreted within the context of a few inherent limitations. First, our sample size was relatively small which limited power; hence, the findings of this study should be replicated in an independent population. It is also important to acknowledge that bisulfite conversion-dependent techniques (850K array and pyrosequencing) cannot distinguish the canonical 5-methylcytosine mark from its oxidized derivative 5-hydroxymethylcytosine (5-hmc), though 5-hmc values tend to be low in placenta [97]. Although pathological examination confirms that the non-aCA preterm samples used in this study were not associated with aCA and/or placental inflammation, it is rarely possible to obtain “normal” placentas from 21 to 37 weeks. Often other placental findings were noted in our non-aCA cases, including placental abruption, spontaneous premature rupture of the membranes, preterm labor, and placenta previa. Multiple etiologies in the non-aCA preterm cases were included to obtain a heterogeneous control population and therefore reduce the likelihood of an association with any given etiology in the non-aCA preterm cases. In addition, though the range of GA overlapped between the aCA and the non-aCA cases, the aCA cases were significantly lower in GA than the non-aCA cases. This is expected, as aCA is associated with preterm birth and therefore the aCA cases were < 37 weeks GA. Because GA-dependent DNAm patterns are observed in the placenta [17], GA was included as an additive covariate in our statistical models. Finally, though aCA is mainly characterized by neutrophil infiltration into the chorioamniotic membranes [20], cellular changes in multiple other cell types including but not limited to Hofbauer cells or trophoblast cells, are also observed in aCA [8]. To fully understand the DNAm variation in aCA-associated placentas, it will be important to study changes in these specific cell populations.

## Conclusion

This work addresses the gaps in our understanding of the epigenetic dynamics associated with aCA, which is linked to an inflammatory response in the newborn. We found aberrant array-wide DNAm associated with aCA placentas and confirmed some of these DNAm changes in an independent set of samples. Although our candidate list of aCA-associated CpG sites may contribute toward identification of diagnostic biomarkers for aCA, further studies with larger sample sizes are required to confirm these placental disease-specific DNAm signatures in maternal blood.

## Additional files

**Additional file 1. Table S1:** is a table listing the demographic, clinical and technical variables for the individual tissue samples in the discovery cohort.** Table S2:** is a table listing the demographic and clinical variables for the independent set of chorionic villus samples in the validation cohort.** Table S3:** is a table listing the probe filtering characteristics for chorionic villi, amnion, and chorion.** Table S4:** is a table listing probes/CpG sites investigated for the biologically relevant candidate CpG site analysis.** Table S5:** is a table listing the probe filtering characteristics for the matched tissue dataset.** Table S6:** is a table listing probes/CpG sites identified as differentially methylated in the discovery cohort (chorionic villi), along with their relevant gene information.

**Additional file 2. Figure S1:** shows the distribution of samples across the ten chips for the discovery cohort.** Figure S2a and S2b:** shows unsupervised hierarchical clustering and PCA on all probes in the 850K array (866,895 CpGs).** Figure S3:** shows the distribution of M values plotted for each pair of technical replicate samples.** Figure S4:** summarizes the primer sequences and reaction conditions used for each pyrosequencing assay.** Figure S5:** shows hierarchical clustering on the 66 differentially methylated CpG sites in chorionic villi.** Figure S6:** shows sex-specific array-wide volcano plots in chorionic villi.** Figure S7:** shows differential methylation at the three candidate CpG sites in males and females.** Figure S8:** shows variation in DNAm between fetal sexes over gestational age.** Figure S9:** shows the distributions of correlation coefficients (R) between DNAm in chorionic villi and fetal membranes compared against the null distribution.** Figure S10:** shows the array-wide volcano plots of differential methylation analysis between the tissue pairs (chorionic villi and amnion; and chorionic villi and chorion).

**Additional file 3. Methods S1:** provides detailed description of data preprocessing of matched tissue dataset.** Methods S2:** provide detailed description on differential methylation analysis on matched tissue comparison dataset.

## Abbreviations

PTB: preterm birth
aCA: acute chorioamnionitis
DNAm: DNA methylation
850K array: Illumina Infinium MethylationEPIC BeadChip
450K array: Illumina Infinium HumanMethylation450 BeadChip
CpG: cytosine–phosphate–guanosine
GA: gestational age
AIM: ancestry informative marker
SNPs: single nucleotide polymorphisms
MDS: multi-dimensional scaling
ICM: inner cell mass
EWAS: epigenome-wide association study
DM: differentially methylated
DMR: differentially methylated region
FDR: false discovery rate
GEO: gene expression omnibus
ns: non-significant
QC: quality control
SD: standard deviation
