# Supplementary material for: DNA methylation profiling of acute chorioamnionitis-associated placentas and fetal membranes: insights into epigenetic variation in spontaneous preterm births
Source: Epigenetics Chromatin. 2018 Oct 29;11:63. doi: 10.1186/s13072-018-0234-9 (PMC6205793; doi:10.1186/s13072-018-0234-9)
Supplement: Supplementary file 2 — Additional file 2. Figure S1: shows the distribution of samples across the ten chips for the discovery cohort. Figure S2a and S2b: shows unsupervised hierarchical clustering and PCA on all probes in the 850K array (866,895 CpGs). Figure S3: shows the distribution of M values plotted for each pair of technical replicate samples. Figure S4: summarizes the primer sequences and reaction conditions used for each pyrosequencing assay. Figure S5: shows hierarchical clustering on the 66 differentially methylated CpG sites in chorionic villi. Figure S6: shows sex-specific array-wide volcano plots in chorionic villi. Figure S7: shows differential methylation at the three candidate CpG sites in males and females. Figure S8: shows variation in DNAm between fetal sexes over gestational age. Figure S9: shows the distributions of correlation coefficients (R) between DNAm in chorionic villi and fetal membranes compared against the null distribution. Figure S10: shows the array-wide volcano plots of differential methylation analysis between the tissue pairs (chorionic villi and amnion; and chorionic villi and chorion). [file 13072_2018_234_MOESM2_ESM.docx]

**Figure S1.** **Distribution of samples across the ten chips for the discovery cohort**. The study included 44 chorionic villous samples (4 run in duplicate for technical controls: replicates), 16 matched amnion samples, and 16 matched chorion samples.


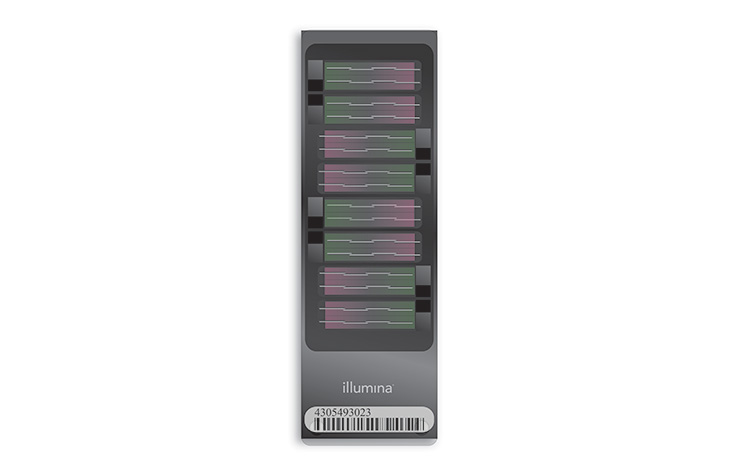


3


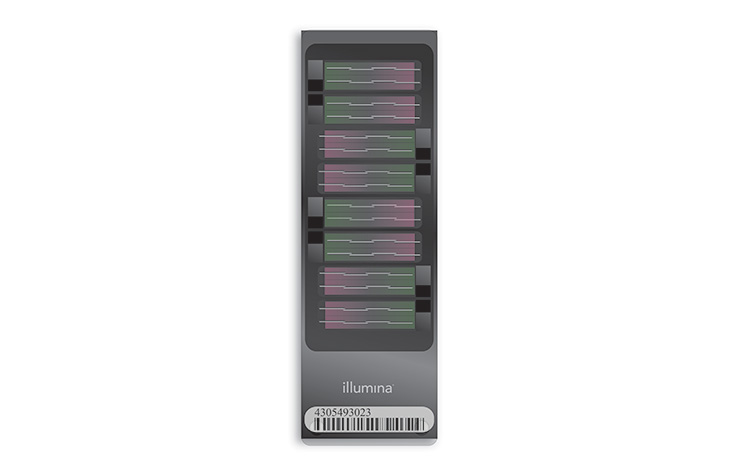


1


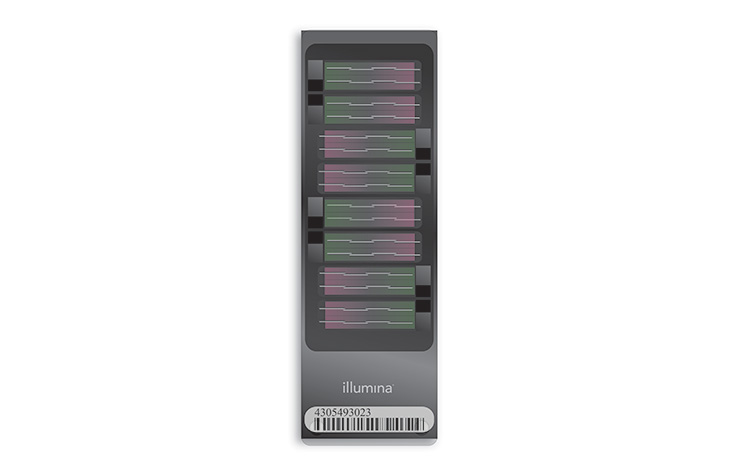


2


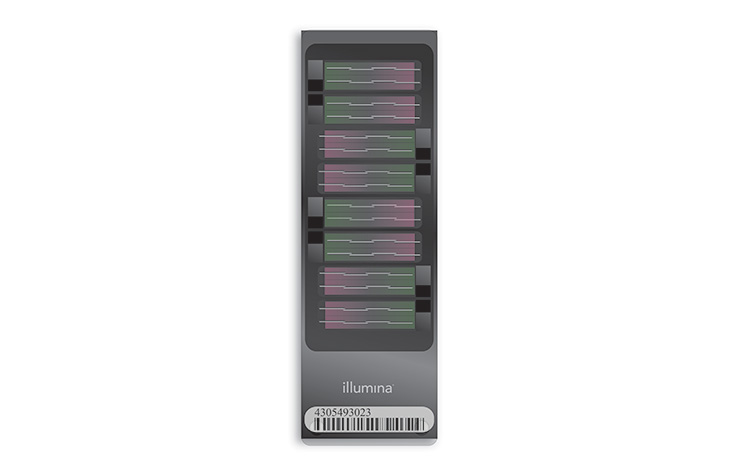


6


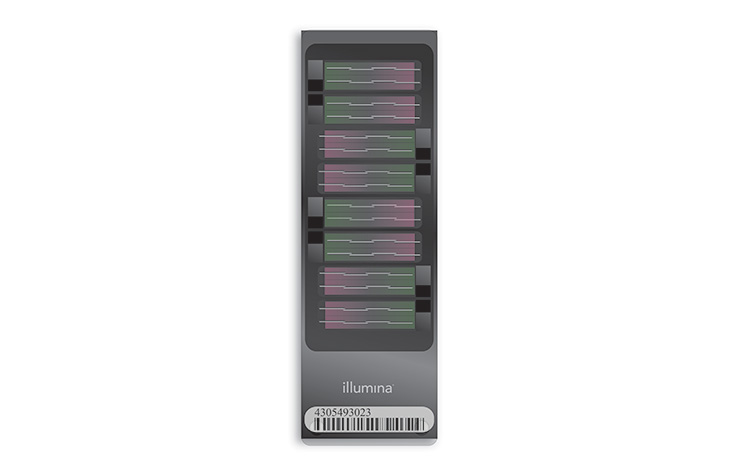


5


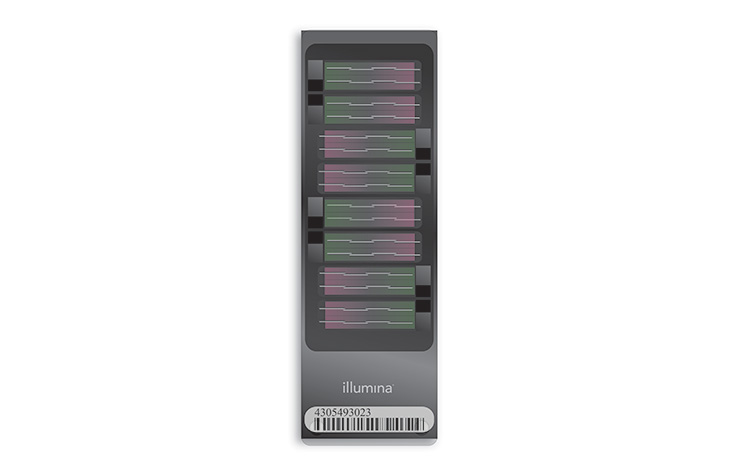


4

Chorionic villi


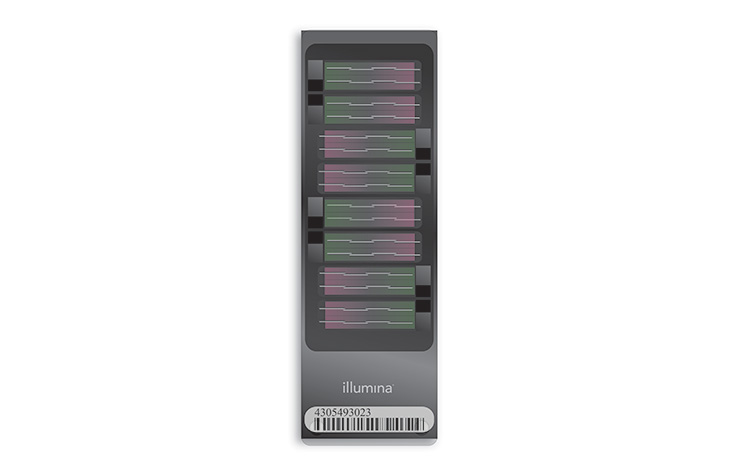


7


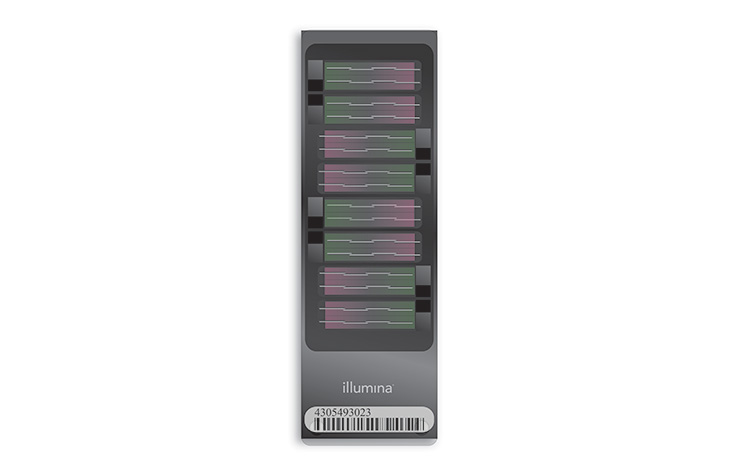


8


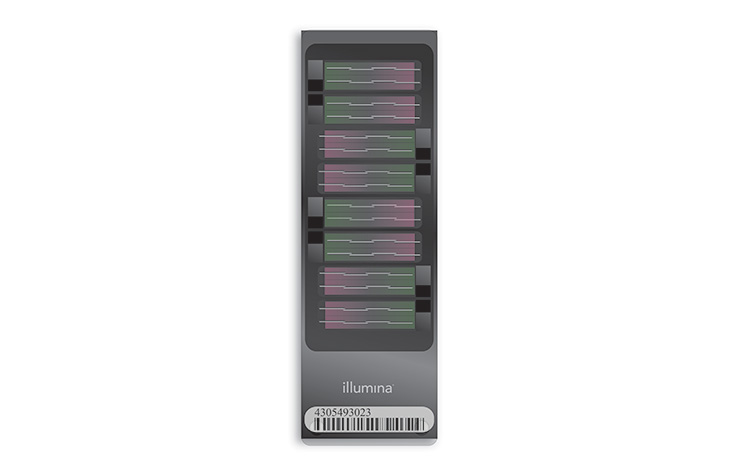


9


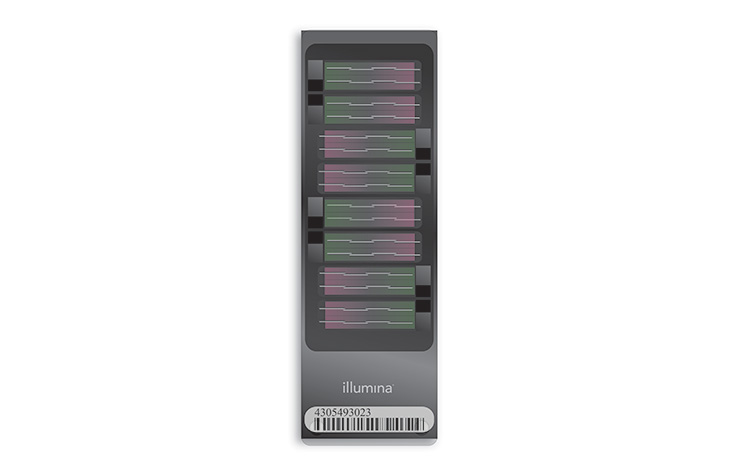


100

**Amnion**

aCA

Non-aCA

Replicate

Replicate

**Chorion**

**Figure S2a and S2b:** **Unsupervised hierarchical clustering and PCA on all probes in the 850k array** (866,895 CpGs). Samples clustered primarily by tissue type (chorionic villi – blue, chorion – green, amnion – red). However, one amnion sample clustered further away from the amnion group (as indicated by the arrow) suggesting it is not representative of its tissue type and was therefore, removed from further analysis.


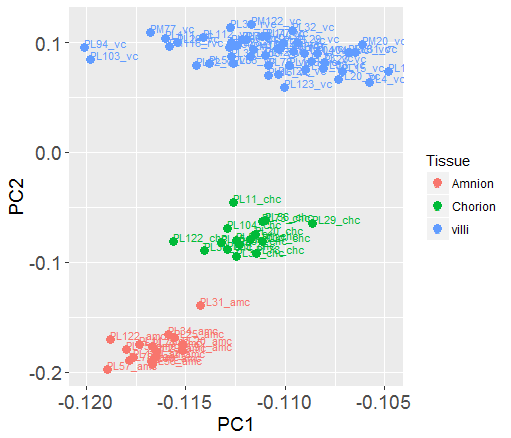

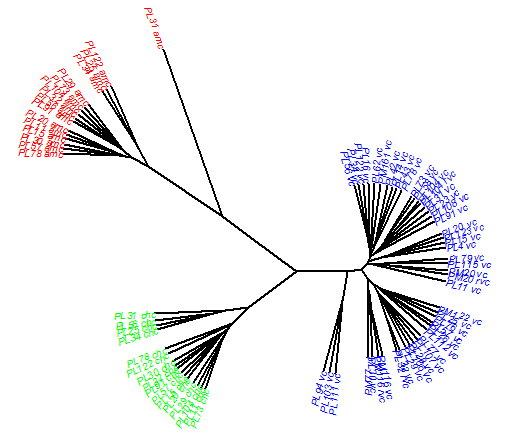


**2a**

**2b**

**Figure S3.** **Distribution of M values was plotted for each pair of technical replicate samples.** The pairwise correlation of probes improved from raw data to batch corrected cleaned data for the four replicates.


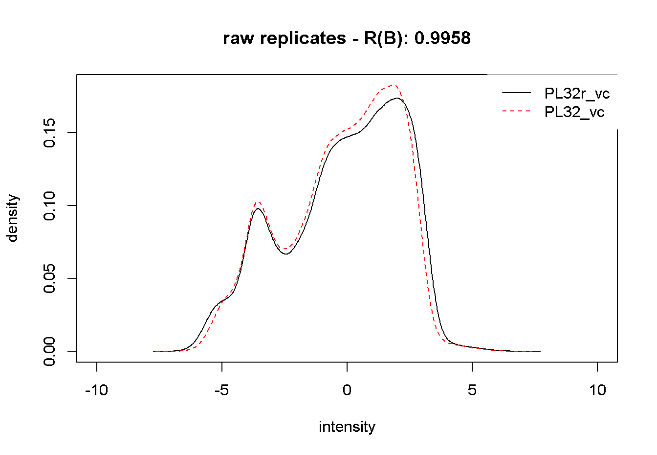

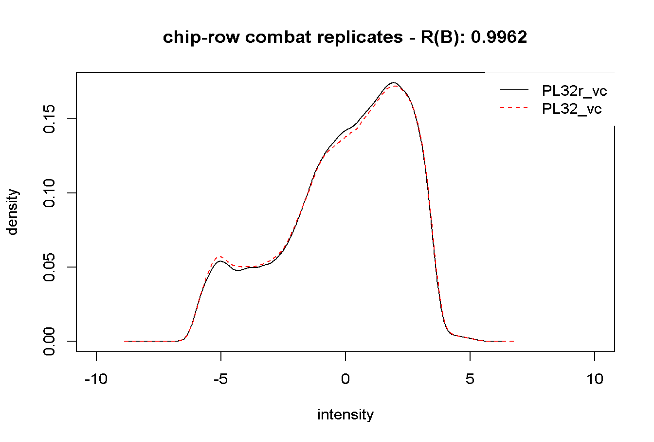

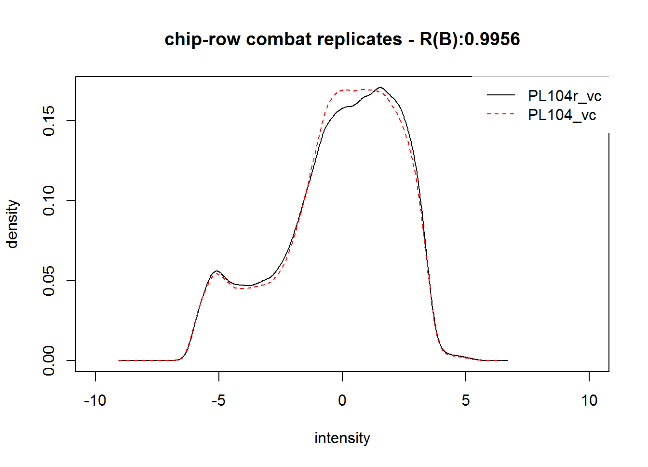

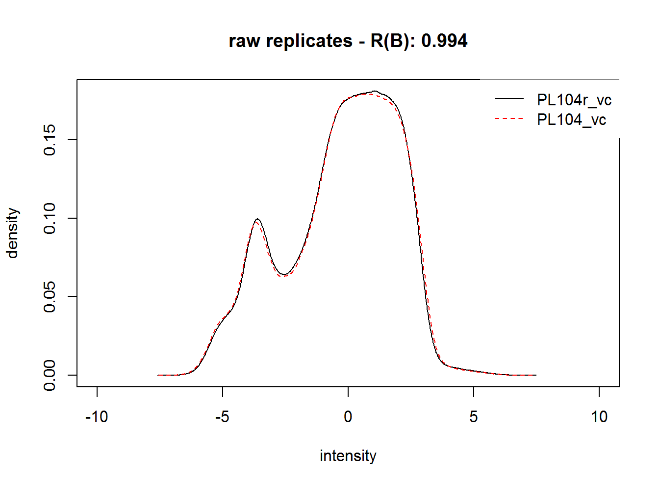

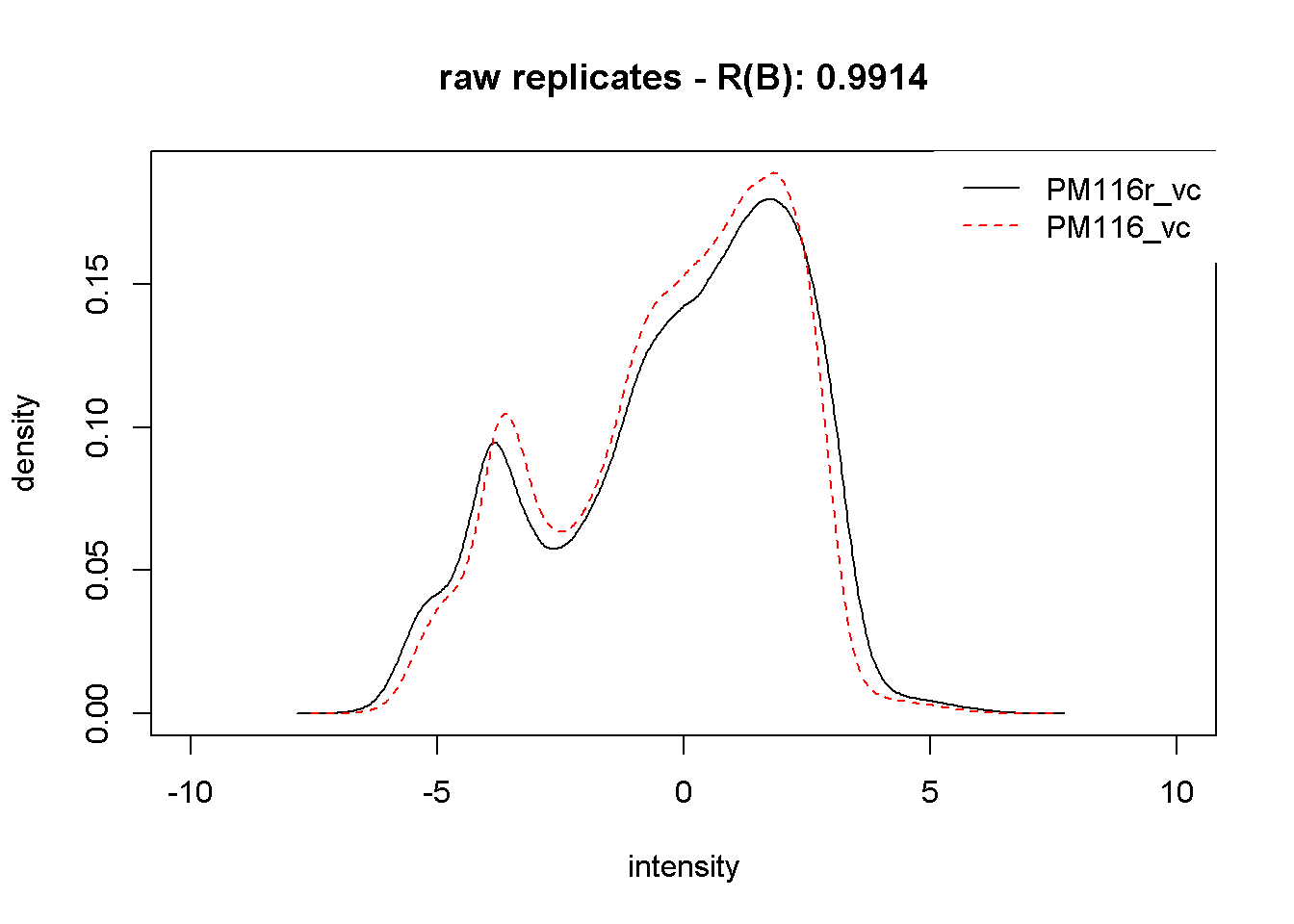

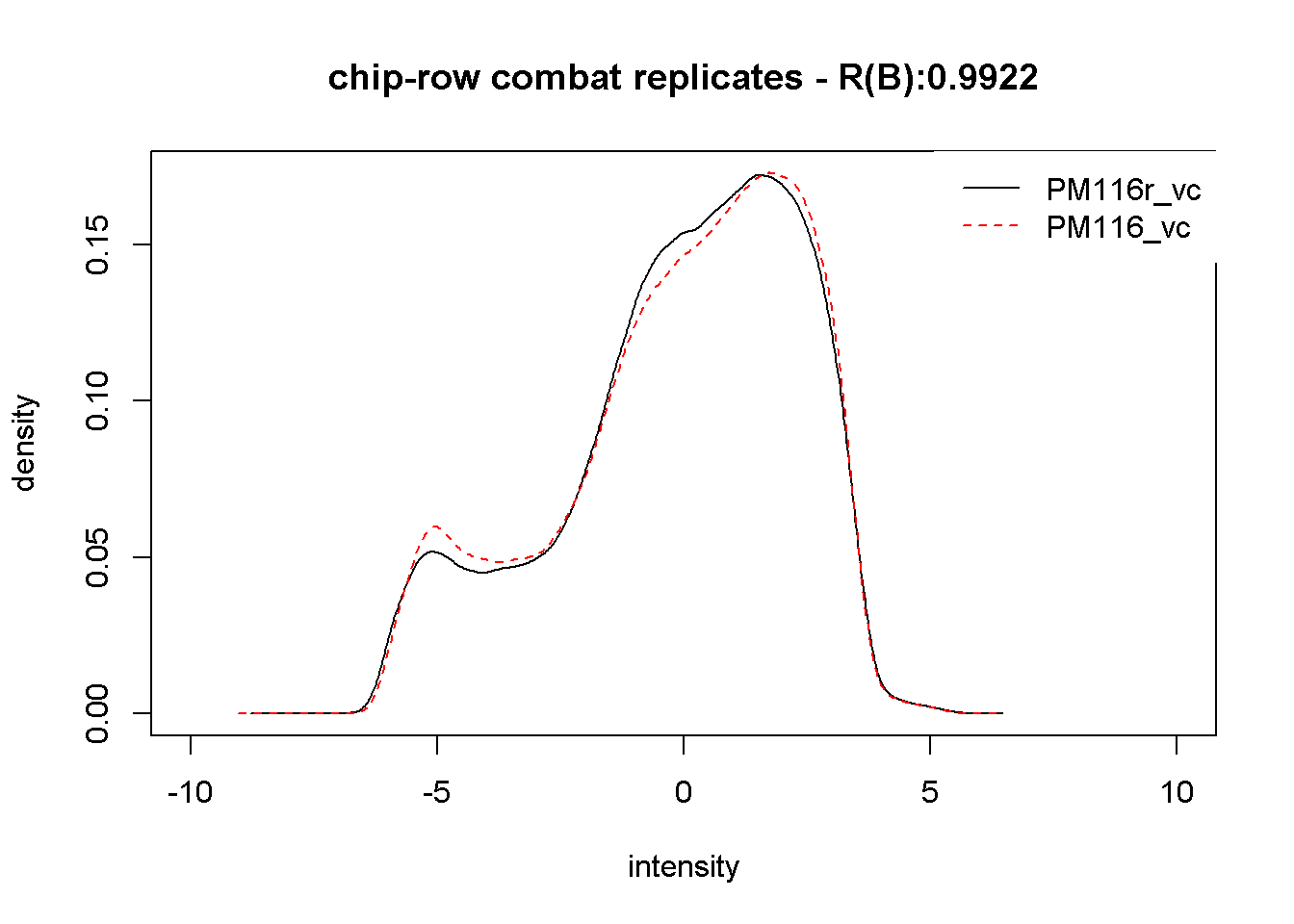

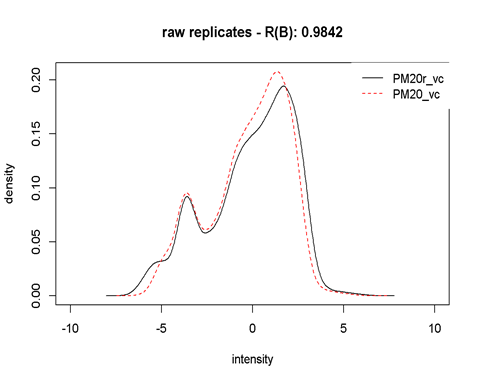

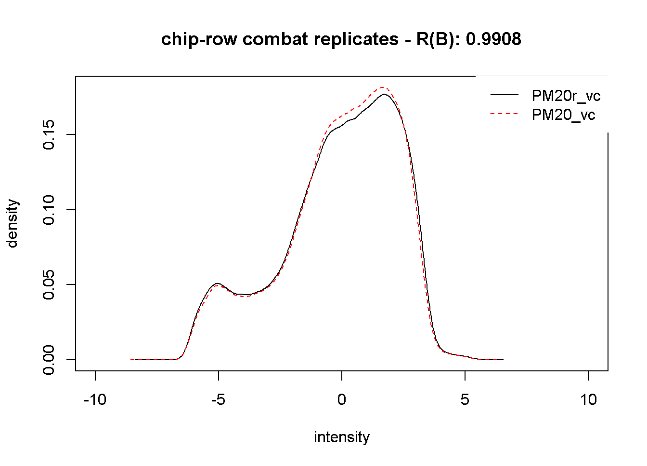


**Raw data r = 0.9958**

**Cleaned data r = 0.9962**

**Raw data r = 0.9940**

**Cleaned data r = 0.9956**

**Raw data r = 0.9914**

**Cleaned data r = 0.9922**

**Raw data r = 0.9842**

**Cleaned data r = 0.9908**

**Figure S4.** **Primer sequences and reaction conditions used for each pyrosequencing assay.**


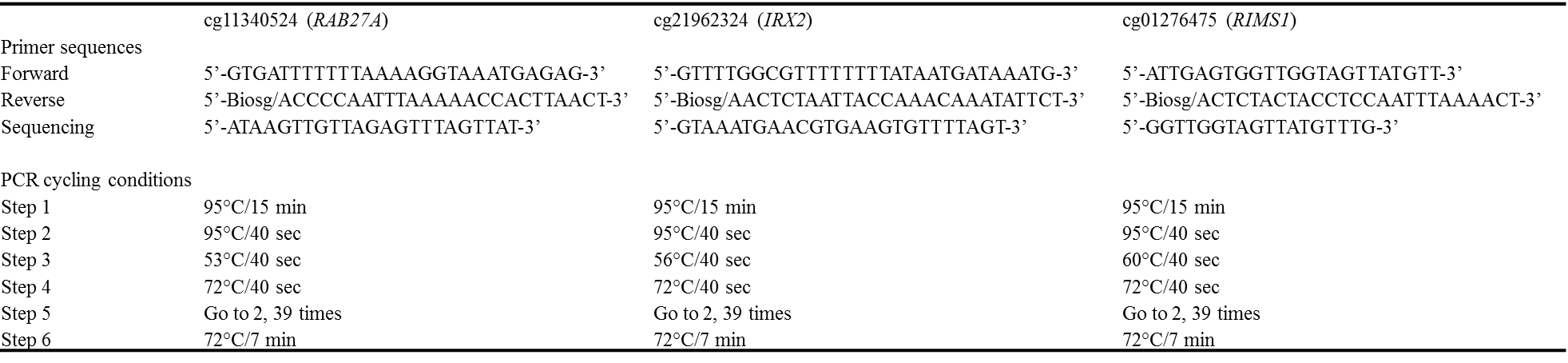


**Figure S5. Hierarchical clustering on the 66 differentially methylated CpG sites in chorionic villi.** Samples clustered by acute chorioamnionitis status. Black labels, non-aCA; blue labels, aCA. * indicates stable and significantly different clusters as determined by pvclust and sigclust2 packages


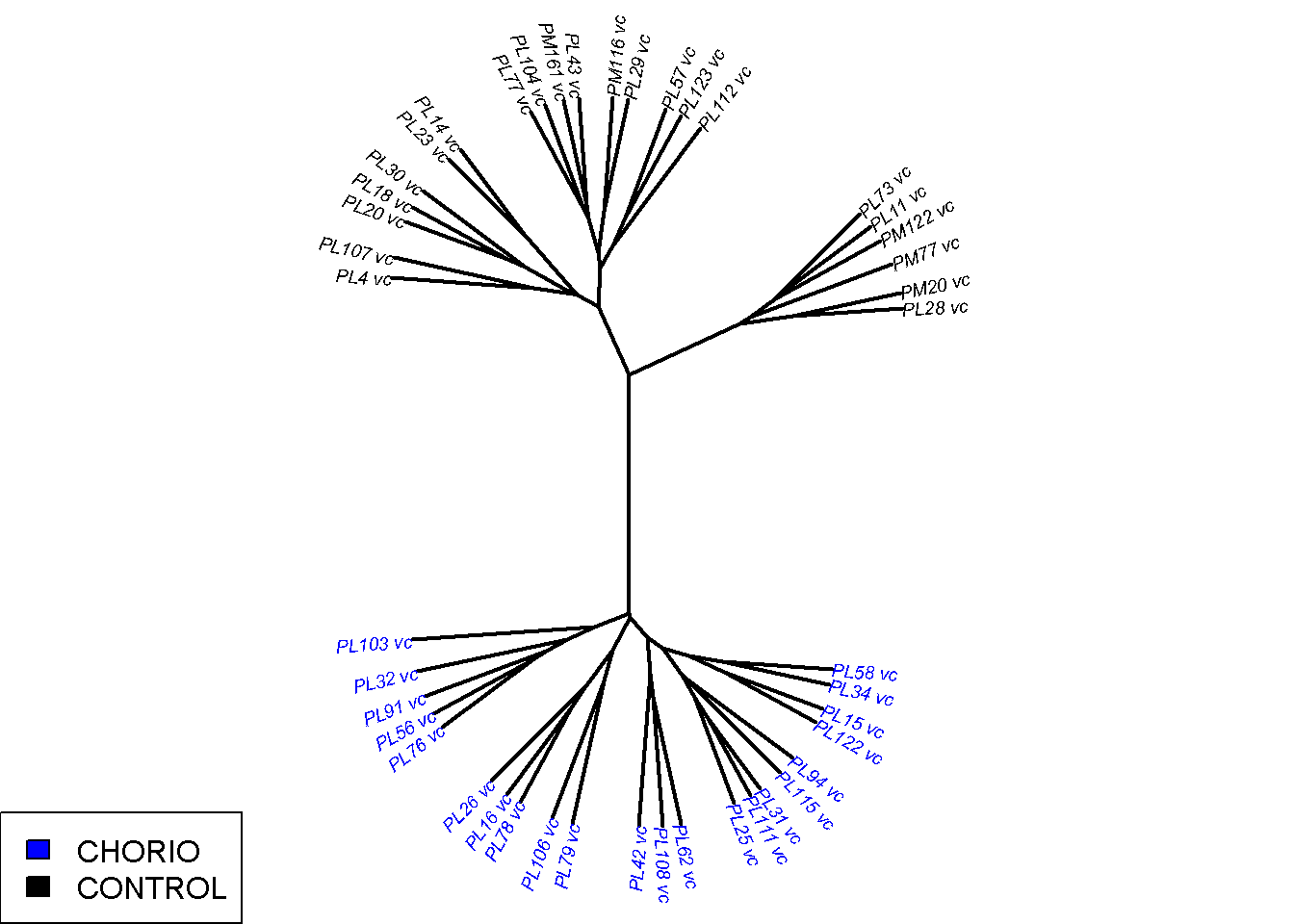


*

*

**Figure S6. Sex-specific array-wide volcano plots in chorionic villi.** For each probe, FDR corrected p-values from fitted linear models were plotted against group differences in DNAm, for males and females separately. The flat volcano plots demonstrate a lack of differential methylation associated with aCA in either of the sexes, after correction for multiple comparisons.

**
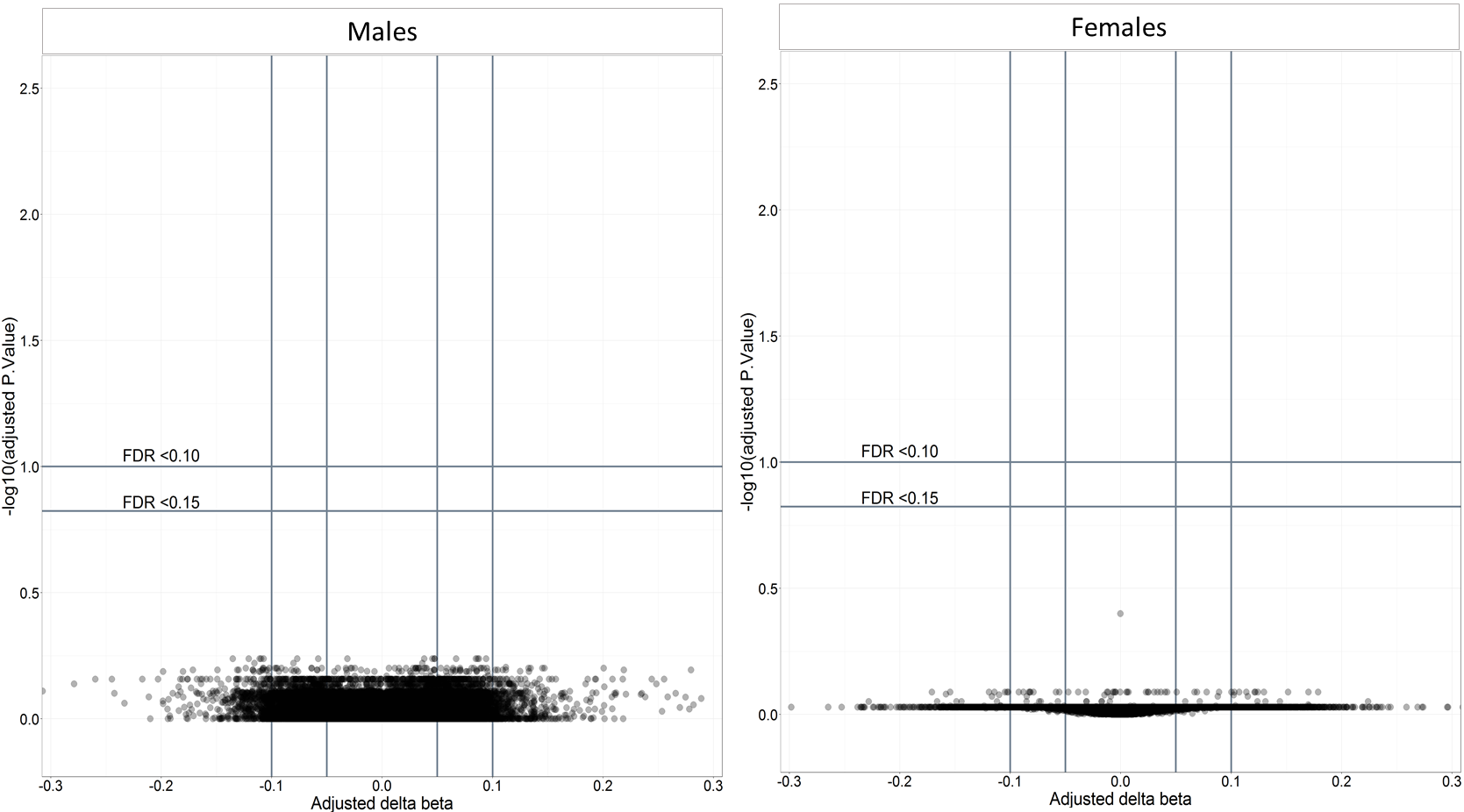
**

**Figure S7. Differential methylation at the three candidate CpG sites in males and females**. In males, we confirmed significant differential DNAm in the discovery cohort and validated in the independent set of samples for cg21962324 and cg11340524. In females, none of the three CpG sites were differentially methylated in the validation cohort, however, similar DNAm trends were observed. % DNA methylation plotted on y-axis was measured by pyrosequencing.


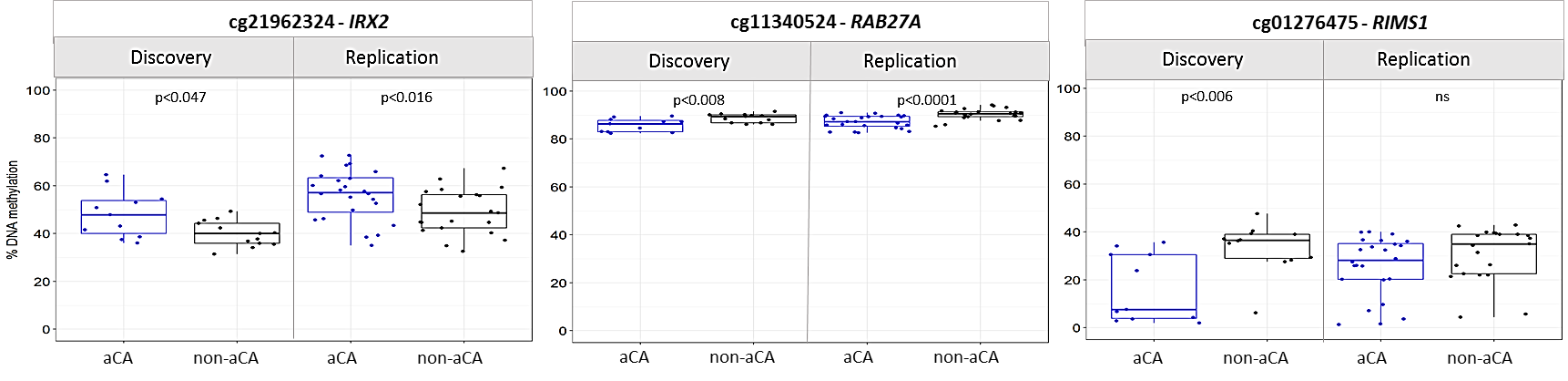

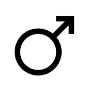

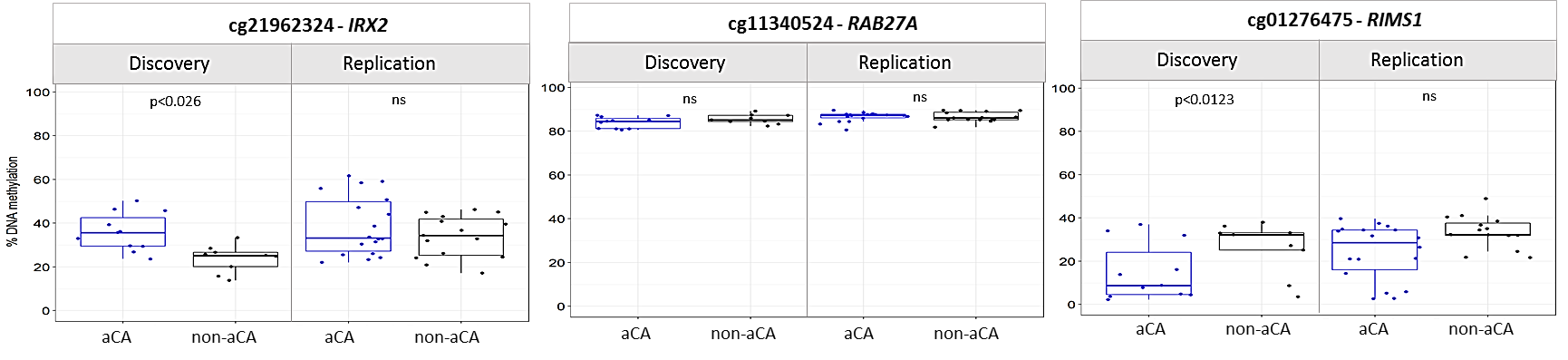

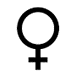


**Figure S8. Variation in DNA methylation between fetal sexes over gestational age**. Sex-specific trends in DNA methylation across gestation was observed for cg21962324 (*IRX2)* and cg01276475 (*RIMS1*). % DNA methylation measured by pyrosequencing is on the y-axis and gestational age (weeks) is on the x-axis.

**
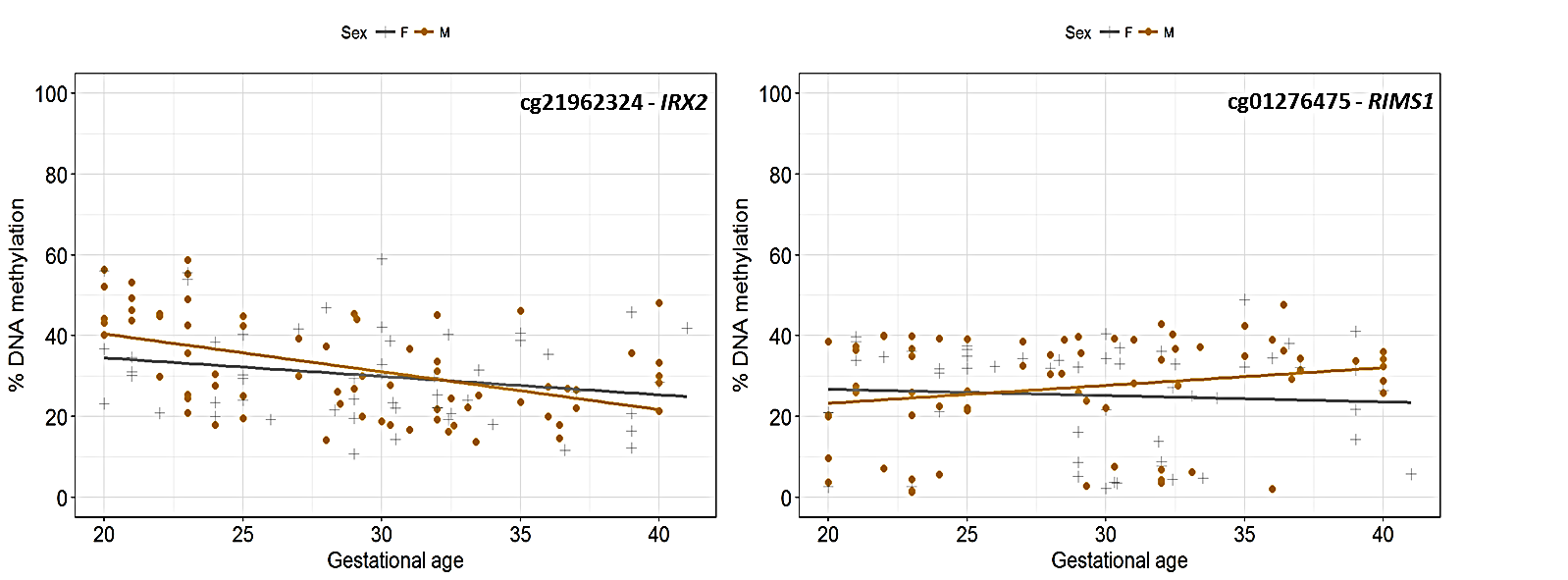
**

**Figure S9. Distributions of correlation coefficients (R) between DNAm in chorionic villi and fetal membranes were compared against the null distribution (grey).** Chorionic villi is more similar to chorion than amnion as evident by skewing of the distribution of correlation coefficient (R) towards stronger positive correlations. As expected, positive skewing was most pronounced when correlations were run between the fetal membranes as both chorion and amnion have a common inner cell mass origin.


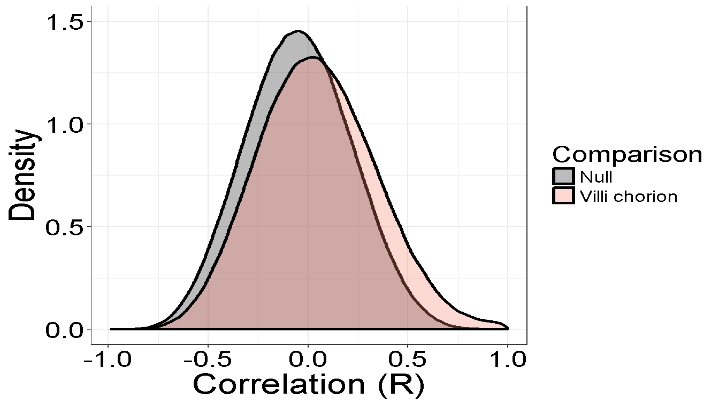


Chorionic villi - Chorion


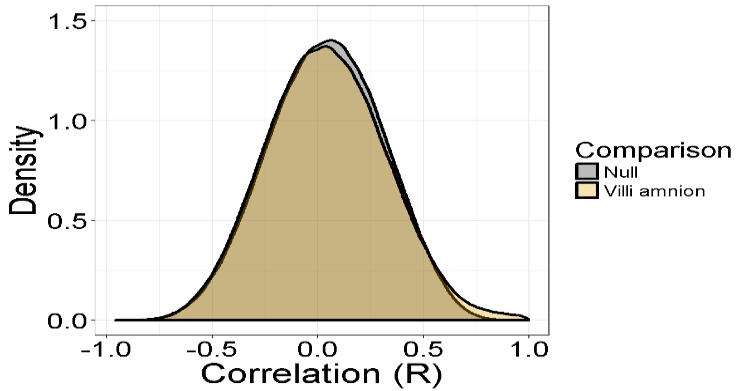


Chorionic villi - Amnion


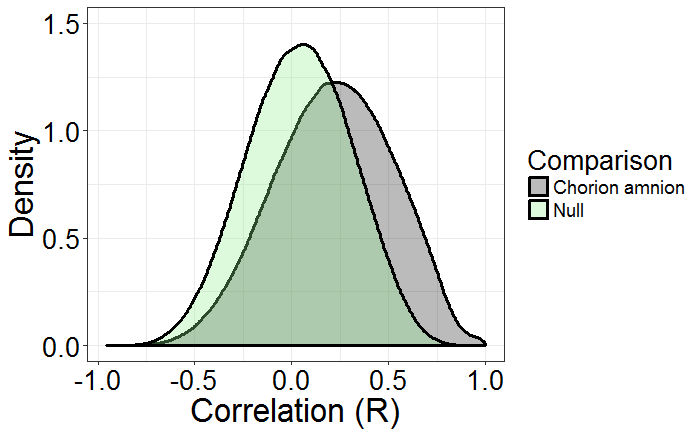


Chorion - Amnion

**Figure S10. Array-wide volcano plots of the differential methylation analysis between the tissue pairs (chorionic villi and amnion; and chorionic villi and chorion).** The x-axis indicates a DNAm difference (Δβ) between the compared tissues. The y-axis represents FDR corrected p-value. Points are colored to highlight CpGs sites that met biological (Δβ > 0.20) and statistical thresholds (FDR < 0.01). Sites highlighted in red are those that are hypermethylated in chorionic villi compared to amnion/chorion. Sites highlighted in blue are those that are hypomethylated in chorionic villi compared to amnion/chorion.


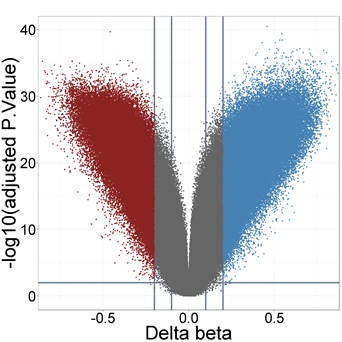


**Chorionic villi - Amnion**


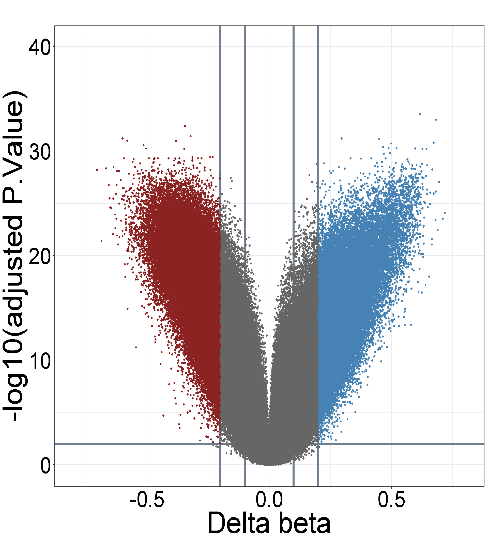


**Chorionic villi - Chorion**
