# Supplementary material for: DNA methylation profiling of acute chorioamnionitis-associated placentas and fetal membranes: insights into epigenetic variation in spontaneous preterm births
Source: Epigenetics Chromatin. 2018 Oct 29;11:63. doi: 10.1186/s13072-018-0234-9 (PMC6205793; doi:10.1186/s13072-018-0234-9)
Supplement: Supplementary file 3 — Additional file 3. Methods S1: provides detailed description of data preprocessing of matched tissue dataset. Methods S2: provide detailed description on differential methylation analysis on matched tissue comparison dataset. [file 13072_2018_234_MOESM3_ESM.docx]

**Method S1 Data preprocessing of matched tissue cohort**

15 samples with matched chorionic villi, amnion and chorion were identified for the matched tissue comparison analysis. Probe filtering was conducted as shown in **Table 3 in Additional file 1**. Functional normalization was used to account for type I- type II probe bias on the 850K array (1). Because the primary objective of this study was to characterize DNAm signatures between aCA cases and controls within a tissue, samples were not randomized by tissue type. It is also important to acknowledge that some of the tissue-specific DM sites may be associated with batch as each tissue type was run on separate 850k chips, but we don’t expect this to affect the result for one comparison more than the other. Additionally, we anticipate tissue-associated DNAm effects to be of larger magnitude than any other technical batch effects.

**Method S2 Differential methylation analysis on matched tissue comparison dataset (Question1)**

To quantify the similarity between fetal membranes and placental chorionic villi, differential DNAm analysis on a per CpG level was performed between the tissue pairs (chorionic villi-chorion and chorionic villi-amnion) by applying a linear model to M values using *limma* (2). In modeling DNAm, tissue type was considered as the main effect and GA, fetal sex, and ancestry were included as covariates. Because each tissue type was collected from the same individuals it is likely that DNAm may be influenced by inter-individual differences. To account for this, the model included a within-individual consensus correlation value estimated by the duplicateCorrelation( ) function in *limma*. Multiple test correction was done on the nominal p values of each tissue pair comparison, using Benjamini and Hochberg false detection rate (FDR) method (3). M values corrected for the additive covariates were logit-transformed to β values and average DNAm for every CpG site was calculated by obtaining the mean β value for each of the tissue type. Magnitude of DNAm differences (Δβ) between tissues was then calculated. Differentially methylated sites were identified based on i) statistical significance (FDR <0.01); and ii) biological significance (Δβ >0.2).

**References**

1. Fortin J, Labbe A, Lemire M, Zanke BW, Hudson TJ, Fertig EJ, et al. Functional normalization of 450k methylation array data improves replication in large cancer studies. Genome biology. 2014;15(11):503.

2. Smyth GK. Limma. linear models for microarray data. In: Bioinformatics and Computational Biology Solutions Using R and Bioconductor. New York: Springer; 2005. 397-420.

3. Yoav Benjamini, Yosef Hochberg. Controlling the False Discovery Rate: A Practical and Powerful Approach to Multiple Testing. Journal of the Royal Statistical Society. Series B (Methodological). 1995 ;57(1):289-300.
